# Diverse Phenotypes, Consistent Treatment: A Study of 30 997 South Asian and White Inflammatory Bowel Disease Patients Using the UK Inflammatory Bowel Disease BioResource

**DOI:** 10.1093/ecco-jcc/jjae186

**Published:** 2024-12-07

**Authors:** Sharmili Balarajah, Laura Martinez-Gili, James Leslie Alexander, Benjamin Harvey Mullish, Robert William Perry, Jia V Li, Julian Roberto Marchesi, Miles Parkes, Timothy Robin Orchard, Lucy Charlotte Hicks, Horace Richard Timothy Williams

**Affiliations:** Department of Metabolism, Digestion and Reproduction, Imperial College London, London, UK; Department of Gastroenterology and Hepatology, Imperial College Healthcare NHS Trust, London, UK; Department of Metabolism, Digestion and Reproduction, Imperial College London, London, UK; Department of Metabolism, Digestion and Reproduction, Imperial College London, London, UK; Department of Gastroenterology and Hepatology, Imperial College Healthcare NHS Trust, London, UK; Department of Gastroenterology, London North West University Healthcare Trust, London, UK; Department of Metabolism, Digestion and Reproduction, Imperial College London, London, UK; Department of Gastroenterology and Hepatology, Imperial College Healthcare NHS Trust, London, UK; Department of Metabolism, Digestion and Reproduction, Imperial College London, London, UK; Department of Gastroenterology and Hepatology, Imperial College Healthcare NHS Trust, London, UK; Department of Metabolism, Digestion and Reproduction, Imperial College London, London, UK; Department of Metabolism, Digestion and Reproduction, Imperial College London, London, UK; Department of Medicine, University of Cambridge, Cambridge, UK; Department of Gastroenterology, Cambridge University Hospitals NHS Trust, Cambridge, UK; Department of Metabolism, Digestion and Reproduction, Imperial College London, London, UK; Department of Gastroenterology and Hepatology, Imperial College Healthcare NHS Trust, London, UK; Department of Metabolism, Digestion and Reproduction, Imperial College London, London, UK; Department of Gastroenterology and Hepatology, Imperial College Healthcare NHS Trust, London, UK; Department of Metabolism, Digestion and Reproduction, Imperial College London, London, UK; Department of Gastroenterology and Hepatology, Imperial College Healthcare NHS Trust, London, UK

**Keywords:** Crohn’s disease, ulcerative colitis, South Asian, ethnicity, phenotype, treatment, IBD BioResource

## Abstract

**Background:**

Studies in the UK and North America have suggested a distinct disease profile in South Asians compared to that of White populations. Disparities in the medical and surgical management of IBD in minority ethnic groups (including Black Americans and Asians) in the US have been shown, while data from Europe, including the UK, have been lacking. This study sought to evaluate South Asian (SA) and White (WH) inflammatory bowel disease (IBD) phenotypes, and to explore treatment approach variations between these cohorts in the UK using the IBD BioResource database.

**Design:**

Differences between WH and SA IBD patients were analysed using demographic, phenotypic and outcome data. Drug utilisation patterns and surgical outcomes were assessed in propensity score-matched (PSM) cohorts with multivariable logistic regression, Cox regression and Kaplan-Meier analysis.

**Results:**

30,997 eligible patients were included. UC was the predominant disease subtype in SA (p<0.001). SA were younger at diagnosis (p<0.001), had a male preponderance (p<0.001), and were less likely to have a smoking history at diagnosis. The SA CD phenotype differed from WH, with less ileal (SA 30.3%, WH 38.4%, p=0.008) and stricturing (SA 16.9%, WH 25.6%, p<0.001) disease, but more perianal disease (SA 38.5%, WH 32.2%, p=0.009). More SA UC patients had extensive disease (SA 41.7%, WH 34.1%, p<0.001). In PSM cohorts, comparing treatments, there were no differences in 5-aminosalicylate, corticosteroid, thiopurine, anti-TNF or vedolizumab use. Survival analysis in matched cohorts showed no difference in time to surgery (CD) or colectomy (UC), and SA ethnicity was not associated with a difference in risk of surgery/colectomy.

**Conclusion:**

Demographic and phenotypic differences exist between UK SA and WH IBD patients, highlighting distinct ethnicity-related variance, and the need for a research focus on under-represented populations. In comparing matched SA and WH patients, no disparity in medical and surgical IBD therapy in UK healthcare has been demonstrated: treatment is consistent regardless of ethnicity.

## 1. Introduction

Current knowledge of the natural history of inflammatory bowel disease (IBD) has been predominantly derived from White (WH) populations.^1^ The ethnic diversity of the UK population is growing, and minority ethnic groups are forecast to make up 20% of the UK population by 2051.^2^ South Asian (SA) now represent the second largest ethnic group within the UK and Canada, after WH groups,^3,4^ and are projected to form one of the largest immigrant populations within the United States by 2026.^5^

Despite the shift in population demographics in Western countries, there remains a paucity of IBD research in non-WH ethnic groups. Studies of the incidence and prevalence of IBD in the SA population in the West have been limited by the extent of the geographic area surveyed and relatively small sample sizes.^6^ A histological US-based study in 2015, from over a million ileocolonoscopies conducted over a 5-year period, stratified IBD according to ancestry: Indians were found to have the highest prevalence of ulcerative colitis (UC) and the third highest prevalence of Crohn’s disease (CD).^7^ In the UK, Misra et al. conducted a prospective study across 5 catchment areas and demonstrated a significantly higher age-adjusted incidence of UC in Indians and Pakistanis compared to WH.^8^

Studies of IBD phenotype in SA IBD patients residing in Europe and North America have been inconclusive, with some contradictory results.^6,8,9^ A cross-sectional study conducted across 5 centers in the UK compared the phenotypic details of 273 SA with 188 Northern European UC patients, demonstrating a higher prevalence of pancolitis in SA.^9^ However, a retrospective cohort study from the US in which SA were age- and sex-matched to WH UC patients found no significant difference.^10^

Aside from phenotypic differences, studies have suggested inequalities in the standardised medical and surgical management of IBD in ethnic minorities in the US. A single-center cross-sectional study concluded that Black (BL) Americans had a lower likelihood of receiving Infliximab for CD, and immunomodulators for UC, compared to WH patients.^11^ Another multicenter cohort study demonstrated that WH IBD patients were twice as likely to be treated with biologics as Asians.^12^ Small US studies comparing the incidence of surgery for CD in SA and WH populations have revealed conflicting results.^10,13^ Investigations into the provision of medical and surgical therapy in IBD across ethnically diverse groups in the UK and Europe have been scarce.

The UK IBD BioResource (IBD-BR) is an open-access national platform that consecutively recruits patients with IBD across 118 centers in the UK. Comprehensive demographic, phenotypic, and treatment data are collected, and regular data curation processes are conducted with subsequent independent reassessment of the data.^14^ The IBD-BR provides an opportunity to explore disease and treatment across diverse groups at an unprecedented scale within a publicly funded healthcare system.

In this study, the IBD-BR dataset was used to characterise demographic and phenotypic variations between WH and SA IBD and to elucidate whether there is ethnicity-related variation in treatment approaches in the UK.

## 2. Methods

### 2.1. Database

This analysis used prospectively collected data from the IBD-BR across 118 UK centres. All participants of the IBD-BR (REC reference 15/EE/0286, IRAS 173561) provided signed consent. An application for data access was approved by the NIHR BioResource Steering Committee. A total of 35 082 individuals with confirmed IBD had been enrolled from the establishment of the database (2016) to the date of data lock and extraction (May 12, 2023).

Structured demographic and phenotypic data were collected at the time of enrollment using a combination of clinical notes review, patient interview, and patient completion of a health and lifestyle questionnaire. Regular data validation procedures, including independent reevaluation of the gathered data, ensure that the database remains contemporaneous.^14^ Unselected consecutive patients were recruited.

### 2.2. Eligibility criteria

**Figure 1:**
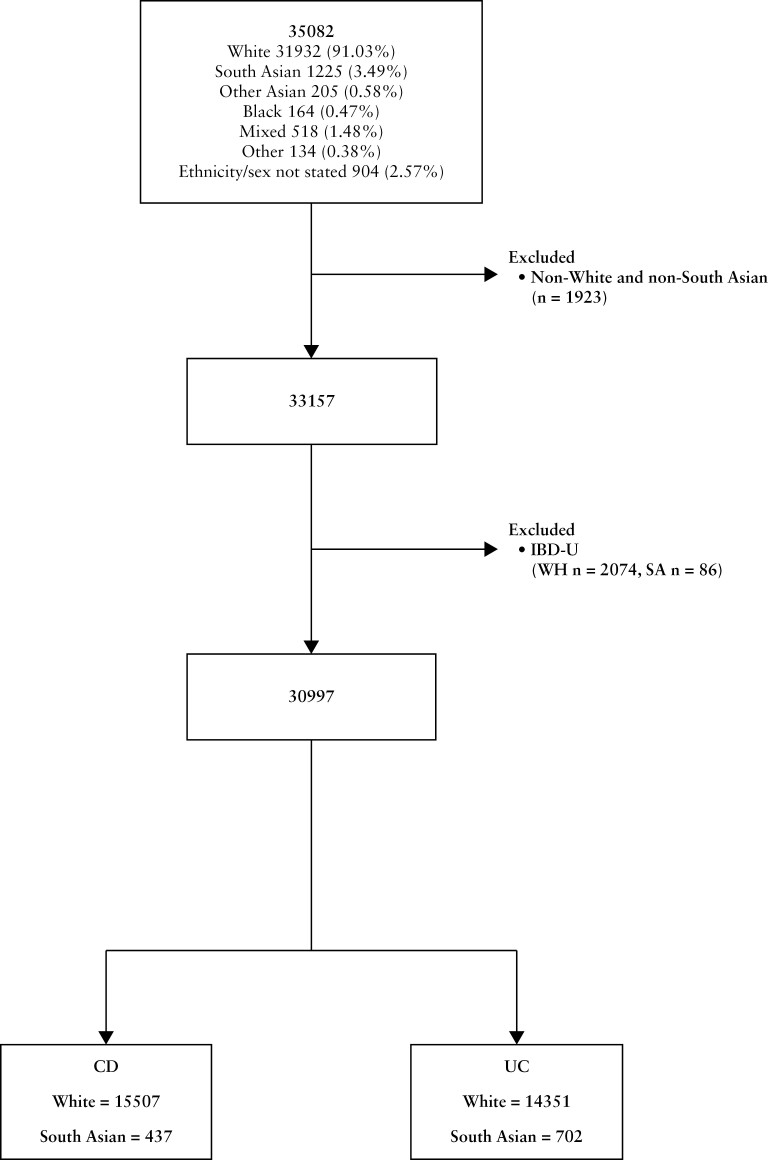
Study flow chart (created in *BioRender.com*).

Patients with confirmed IBD and of SA (Bangladeshi, Indian, or Pakistani) or WH ethnicity, collected as per the Goverment Statistical Service  harmonised standard,^15^ were included. Patients from other ethnic groups or with IBD-unclassified (IBD-U) were excluded from the analysis in view of the smaller numbers (Figure 1).

### 2.3. Statistics

All analyses were conducted in R (version 4.3.0).^16^ Descriptive statistics were used to summarise demographic and clinical characteristics. Chi-squared and Mann-Whitney U tests were used to analyse categorical and continuous variables, respectively. Chi-squared post hoc testing of the standardised residuals was done for significant categorical variables with more than 2 classes, and P values adjusted for multiple comparisons (p_adj_) using Benjamini-Hochberg.

#### 2.3.1. Medication use

Differences in the use of medications were assessed using logistic regression and adjusted for standardised age at diagnosis, sex, smoking history at time of diagnosis, disease duration, disease location (CD) or extent (UC), disease behavior (CD), and perianal disease (CD) (Supplementary text; Supplementary Tables 5 and 6). For each medication type, only patients who were diagnosed in or after the year in which the drug was approved were included for comparison (corticosteroids and aminosalicylates, 1960^17^; thiopurines, 1980^18^; anti-tumor necrosis factor agents [anti-TNFs], 1999 [CD]^19^ and 2008 [UC]^20^; vedolizumab, 2015^21,22^). Anti-TNFs included infliximab, adalimumab, and golimumab. Ustekinumab use (UK approved in 2017) could not be assessed due to the small sample size of SA treated with the drug prior to matching (CD *n* = 37, UC *n* = 1).

#### 2.3.2. Surgery

Surgery was defined as previous intestinal resection, and perianal intervention was specifically assessed in those with perianal involvement. Differences in the time from diagnosis to surgery were assessed using Kaplan-Meier and log-rank test, and Cox regression adjusting for standardised age at diagnosis, sex, smoking history at time of diagnosis, disease location (CD) or extent (UC), disease behavior (CD), perianal disease (CD), and use of medications (corticosteroids, thiopurines, and biologics) (Supplementary text; Supplementary Table 7). As above, the analysis did not account for the time from diagnosis to consent due to its colinear relationship with age at diagnosis.

### 2.4. Generation of matched subsets

Medication use and time to surgery were assessed in subsets of WH and SA with similar characteristics to specifically assess whether ethnicity influences the provision of medication and surgery in phenotypically similar cohorts. Groups were matched based on age at diagnosis, diagnostic era (pre-thiopurines [diagnosed before 1980], thiopurines [diagnosed between 1980 and 1998 for CD and 2007 for UC], and biologic [diagnosed from 1999 for CD and 2008 for UC]), disease duration, sex, smoking status, and Montreal disease classification (disease location [CD], disease extent [UC], disease behavior [CD], and perianal disease [CD]). For medications, patients diagnosed before the relevant drug inception year were excluded, as previously discussed. Propensity score (PS) matching was conducted using nearest neighbor matching with replacement, using the MatchIt-R package.^23^ The standardised mean difference (SMD) was used to check for imbalance, which was defined as a value of > 0.1.^24^ Almost all variables showed an SMD < 0.1 after matching (Supplementary Figures 1 and 2). In cases where the SMD was > 0.1, sensitivity analyses were conducted with the exclusion of relevant categories/subgroups as indicated in each case.

## 3. Results

There was a total of 33 321 patients of WH, SA, and BL patients with confirmed IBD. A difference in IBD subtype was observed between the ethnic groups (Supplementary Tables 1 and 2): CD was more common in WH and BL (WH 48.29%; BL 47.56%; SA 35.67%, *p* < 0.001), and UC was more common in SA (SA 57.31%; WH 45.25%; BL 44.51%, *p* < 0.001). Similar proportions were observed in the IBD-U cohort (WH 6.46%, SA 7.02%; BL 7.93%). Due to their comparatively limited number, BL patients were excluded from the subsequent analyses. A total of 15 944 CD (SA *n* = 437; WH *n* = 15 507) and 15 053 UC (SA *n* = 702; WH *n* = 14 531) patients were further analysed to explore differences in phenotype and disease management (Figure 1).

### 3.1. Demographics

#### 3.1.1. Crohn’s disease

A male preponderance was observed in SA, while the reverse was seen in WH (males: SA 63.4%; WH 45.3%; *p* < 0.001; Table 1). SA were slightly younger at diagnosis {SA 24 (interquartile range [IQR] 17-36); WH 26 (19-39) years; *p* < 0.001} and less likely to have a smoking history at diagnosis (never smoker: SA 72.7%; WH 46.1%; p_adj_ < 0.001). Similar trends were seen (male preponderance and lower prevalence of smokers) in assessing WH vs SA subgroups (Bangladeshis, Indians, Pakistanis; see Supplementary Table 3).

**Table 1:** Demographic and phenotypic characteristics of South Asian and White patients with CD.

	WH N = 15507	SA N = 437	p	p_adj_
**Gender, N (%)**				
Female	8477 (54.7)	160 (36.6)	<0.001^***^	_
Male	7030 (45.3)	277 (63.4)		
**Age (years) at diagnosis, Median (IQR)**	26 (19-39)	24 (17-36)	<0.001^***^	_
**Smoking status at diagnosis, N (%)**			<0.001^***^	
Never smoked	6715 (46.1)	299 (72.7)		<0.001^***^
Ex-smoker	5385 (36.9)	68 (16.5)		<0.001^***^
Current smoker	2475 (17.0)	44 (10.7)		0.002^**^
**Disease duration, N (%)**			<0.001^***^	
Less than 5 years	715 (4.6)	34 (7.8)		0.006^**^
5-9 years	3654 (23.7)	132 (30.3)		0.006
10-14 years	3134 (20.3)	98 (22.5)		0.50
15-19 years	2313 (15.0)	79 (18.2)		0.17
20 years or more	5632 (36.5)	92 (21.1)		<0.001^***^
**Disease location, N (%)**			0.012^*^	
Ileal	5765 (38.4)	124 (30.3)		0.008^**^
Colonic	3972 (26.4)	121 (29.6)		0.41
Ileocolonic	5108 (34.0)	158 (38.6)		0.20
Isolated upper GI	187 (1.2)	6 (1.5)		1.00
**Disease behavior, N (%)**			<0.001^***^	
Non-stricturing, non-penetrating	8975 (61.7)	289 (73.9)		<0.001^***^
Stricturing	3722 (25.6)	66 (16.9)		<0.001^***^
Penetrating	1841 (12.7)	36 (9.2)		0.08
Perianal involvement	4677 (32.2)	153 (38.5)	0.009^**^	
**Extraintestinal manifestations, N (%)**				
Enteropathic arthritis	1162 (8.0)	29 (7.2)	0.62	_
Erythema nodosum	440 (3.0)	9 (2.2)	0.46	_
Iritis	466 (3.2)	7 (1.7)	0.14	_
Orofacial granulomatosis	367 (2.5)	6 (1.5)	0.26	_
Psoriasis	923 (6.3)	19 (4.7)	0.24	_
Ankylosing spondylitis	390 (2.7)	9 (2.2)	0.71	_
Primary sclerosing cholangitis	104 (0.7)	2 (0.5)	0.84	_

^*^ *p* < 0.05.

^**^ *p* < 0.01.

^***^ *p* < 0.001.

Abbreviations: CD, Crohn’s disease; SA, South Asian; WH, White.

#### 3.1.2. Ulcerative colitis

In UC, a male preponderance was also observed in SA (males: SA 58.8%; WH 50.3%; *p* < 0.001; Table 2) while there was an even sex distribution in WH. As in CD, SA were younger at diagnosis (SA 29 [IQR 22-38]; WH 35 [25-48] years, *p* < 0.001) and less likely to have a history of smoking at diagnosis (never smoker: SA 77.5%; WH 46.8%; *p* < 0.001). On subgroup analysis, a male preponderance was seen in Indians; a lower prevalence of smoking history was seen across all three groups (Bangladeshis, Indians, and Pakistanis; Supplementary Table 4).

**Table 2:** Demographic and phenotypic characteristics of South Asian and White patients with UC.

	WH *N* = 14351	SA *N* = 702	p	p_adj_
**Gender, N (%)**				
Female	7131 (49.7)	289 (41.2)	<0.001^***^	_
Male	7220 (50.3)	413 (58.8)		
**Age (years) at diagnosis, Median (IQR)**	35 (25-48)	29 (22-38)	<0.001^***^	_
**Smoking status at diagnosis, N (%)**			<0.001^***^	
Never smoked	6359 (46.8)	510 (77.5)		<0.001^***^
Ex-smoker	6339 (46.7)	113 (17.2)		<0.001^***^
Current smoker	876 (6.5)	35 (5.3)		0.49
**Disease duration, N (%)**			0.002^**^	
Less than 5 years	734 (5.1)	45 (6.4)		0.43
5-9 years	4301 (30.0)	221 (31.6)		0.77
10-14 years	3153 (22.0)	172 (24.6)		0.43
15-19 years	2102 (14.7)	113 (16.1)		0.72
20 years or more	4030 (28.1)	149 (21.3)		<0.001^***^
**Disease extent,** ** N** **(%)**			<0.001^***^	
Proctitis	2184 (17.2)	72 (12.1)		0.004^**^
Left-sided	6178 (48.7)	274 (46.2)		0.47
Extensive	4321 (34.1)	247 (41.7)		<0.001^***^
**Extraintestinal manifestations, N (%)**				
Enteropathic arthritis	558 (4.1)	40 (6.0)	0.02^*^	
Erythema nodosum	93 (0.7)	2 (0.3)	0.35	
Iritis	238 (1.7)	6 (0.9)	0.14	
Orofacial granulomatosis	36 (0.3)	2 (0.3)	1.0	
Psoriasis	522 (3.8)	24 (3.6)	0.83	
Ankylosing spondylitis	211 (1.5)	7 (1.0)	0.38	
Primary sclerosing cholangitis	283 (2.1)	8 (1.2)	0.15	

^*^ *p* < 0.05.

^**^ *p* < 0.01.

^***^ *p* < 0.001.

Abbreviations: SA, South Asian; UC, ulcerative colitis; WH, White.

### 3.2. Phenotype

#### 3.2.1. Crohn’s disease

A difference in disease location was observed, with a lower prevalence of ileal disease in SA (SA 30.3%; WH 38.4%; p_adj_ = 0.008; Table 1). SA were more likely to have perianal involvement (SA 38.5%; WH 32.2%; *p* = 0.009) and less likely to have stricturing disease (SA 16.9%; WH 25.6%; p_adj_ < 0.001) relative to WH patients. There were no differences in the occurrence of extraintestinal manifestations (Table 1). On subgroup analysis, Bangladeshis were found to have a higher prevalence of ileocolonic disease (Bangladeshi 55.4%; WH 34%; *p* < 0.001) and perianal involvement (Bangladeshi 42.9%; WH 32.2%, *p* = 0.049) compared to WH (Supplementary Table 3). Indians had a higher prevalence of inflammatory disease behavior relative to WH (Indians 77.3%; WH 61.7%; *p* < 0.001; Supplementary Table 3).

#### 3.2.2. Ulcerative colitis

A larger proportion of SA had extensive disease (SA 41.7%; WH 34.1%; p_adj_ < 0.001) and a smaller proportion had proctitis (SA 12.1%; WH 17.2%; p_adj_ = 0.004; Table 2). SA were more likely to have enteropathic arthritis (SA 6.0%; WH 4.1%; *p* = 0.02; Table 2). On assessing differences between WH and SA subgroups, Indians and Pakistanis were found to have a higher prevalence of extensive disease (extensive disease: Indian 39.5%, WH 34.1%, *p* = 0.07; Pakistani 47.2%, WH 34.1%, *p* = 0.01; Supplementary Table 4).

### 3.3. Medication use

#### 3.3.1. Propensity-matched analysis in CD

Differences in medication use were assessed between SA and WH in PS-matched groups (see *Methods*), ensuring comparison between phenotypically similar cohorts. Overall, thiopurine, anti-TNF, and vedolizumab were associated with an earlier age at diagnosis (thiopurines, odds ratio [OR] 0.68 [95% CI 0.55-0.84], *p* < 0.001; anti-TNFs, OR 0.58 [95% CI 0.47-0.72], *p* < 0.001; vedolizumab, OR 0.36 [95% CI 0.12-0.89], *p* = 0.045). Anti-TNFs were associated with perianal involvement (OR 3.23 [95% CI 2.14-4.96], *p* < 0.001). Anti-TNFs were additionally associated with ileocolonic disease (OR 1.89 [95% CI 1.21-2.97], *p* = 0.005), and both anti-TNFs and corticosteroids associated with stricturing disease {anti-TNFs, OR 1.77 (95% CI 1.77 [1.02-3.14]), *p* = 0.045; corticosteroids, OR 1.84 [95% CI 1.15-3.01], *p* = 0.01}.

No differences in corticosteroid (OR 1.09 [95% CI 0.79-1.50], *p* = 0.60), thiopurine (OR 1.03 [95% CI 0.71-1.49], *p* = 0.89), anti-TNF (OR 0.83 [95% CI 0.57-1.18], *p* = 0.30), or Vedolizumab (OR 3.61 [95% CI 0.96-17.82], *p* = 0.08) use were found between WH and SA (Table 3).

**Table 3:** Adjusted OR for medication use in White (WH) vs South Asian (SA) IBD patients in propensity-matched cohorts.

	WH (referent)	SA	OR (95% CI)	p
**CD, N (%)**				
** **Corticosteroids	193 (60.5)	211 (63.0)	1.09 (0.79-1.50)	0.60
** **Thiopurines	243 (75.9)	253 (76.0)	1.03 (0.71-1.49)	0.89
** **Anti-TNFs	180 (62.3)	184 (60.3)	0.83 (0.57-1.18)	0.30
** **Vedolizumab	3 (3.3)	9 (9.9)	3.61 (0.96-17.82)	0.08
**UC, N (%)**				
** **5-aminosalicylates	447 (86.5)	465 (84.1)	0.83 (0.59-1.17)	0.29
** **Corticosteroids	334 (64.6)	354 (64.0)	0.97 (0.75-1.26)	0.84
** **Thiopurines	274 (53.9)	297 (54.2)	1.00 (0.78-1.29)	0.98
** **Anti-TNFs	117 (34.6)	130 (35.2)	1.02 (0.74-1.41)	0.89
** **Vedolizumab	21 (14.4)	22 (13.9)	0.94 (0.49-1.82)	0.85

Abbreviations: CD, Crohn’s disease; SA, South Asian; UC, ulcerative colitis; WH, White.

#### 3.3.2. Propensity-matched analysis in UC

Corticosteroids, thiopurines, and biologics were associated with a younger age at diagnosis (corticosteroids, OR 0.80 [95% CI 0.68-0.94], *p* = 0.007; thiopurines, OR 0.67 [95% CI 0.56-0.78], *p* < 0.001; anti-TNFs, OR 0.66 [95% CI 0.53-0.82], *p* < 0.001) and extensive disease (corticosteroids, OR 2.80 [95% CI 1.87-4.22], *p* < 0.001; thiopurines, OR 3.35 [95% CI 2.21-5.15], *p* < 0.001; anti-TNFs, OR 4.76 [95% CI 2.54-9.62], *p* < 0.001; vedolizumab, OR 9.13 [95% CI 1.80-166.78], *p* = 0.03).

No ethnicity-related differences were noted in the use of aminosalicylates (OR 0.83 [95% CI 0.59-1.17], *p* = 0.29), corticosteroids (OR 0.97 [95% CI 0.75-1.26], *p* = 0.84), thiopurines (OR 1.00 [95% CI 0.78-1.29], *p* = 0.98), anti-TNFs (OR 1.02 [95% CI 0.74-1.41], *p* = 0.89), or vedolizumab (OR 0.94 [95% CI 0.49-1.82], *p* = 0.85; Table 3).

### 3.4. Surgery

#### 3.4.1. Propensity-matched analysis in CD

**Figure 2:**
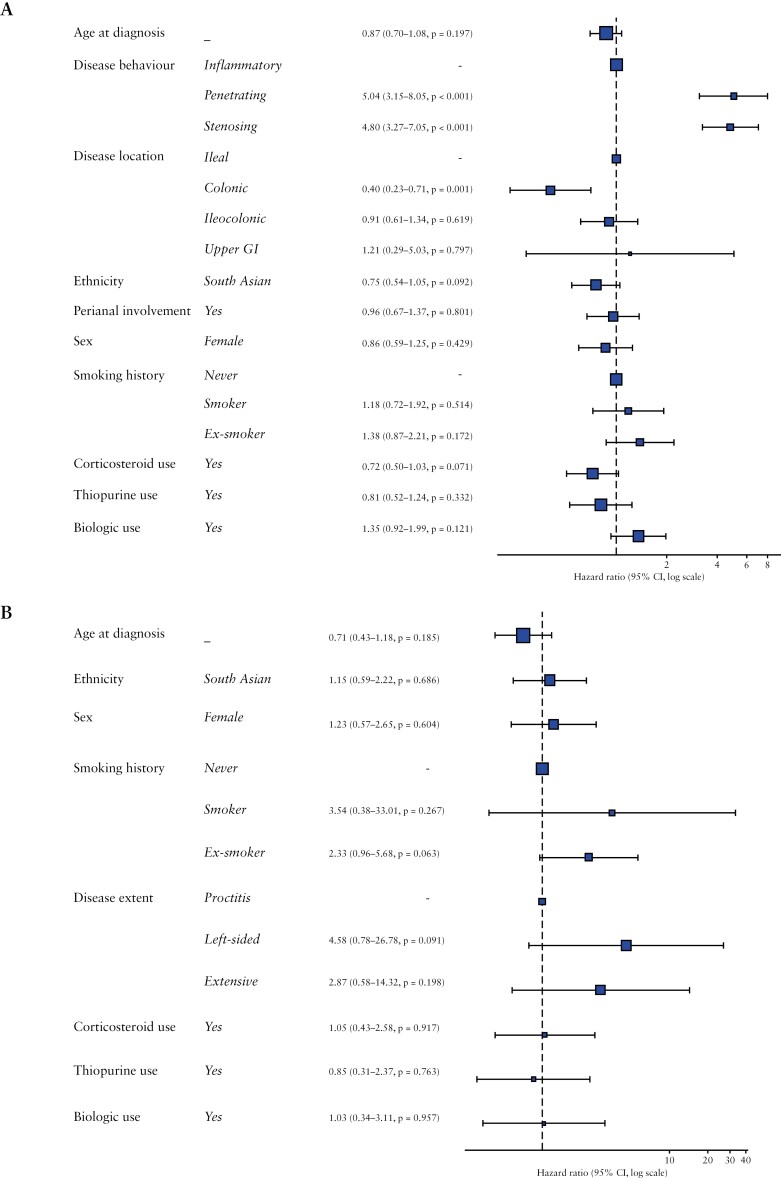
Hazard ratios in propensity-score matched groups: Risk of resection in CD (A), Risk of colectomy in UC (B).

Analysis within matched cohorts was used to assess the risk of having surgery in those with clinically similar phenotypes. In these cohorts, 70 (21.5%) SA and 84 (26.7%) WH underwent a surgical resection. An increased risk of surgery was associated with penetrating (OR 5.04 [95% CI 3.15-8.05], *p* < 0.001) and stricturing (OR 4.80 [95% CI 3.27-7.05], *p* < 0.001) disease, and ileal (relative to colonic) involvement (colonic, OR 0.40 [95% CI 0.23-0.71], *p* = 0.001) (see Figure 2A).

**Figure 3:**
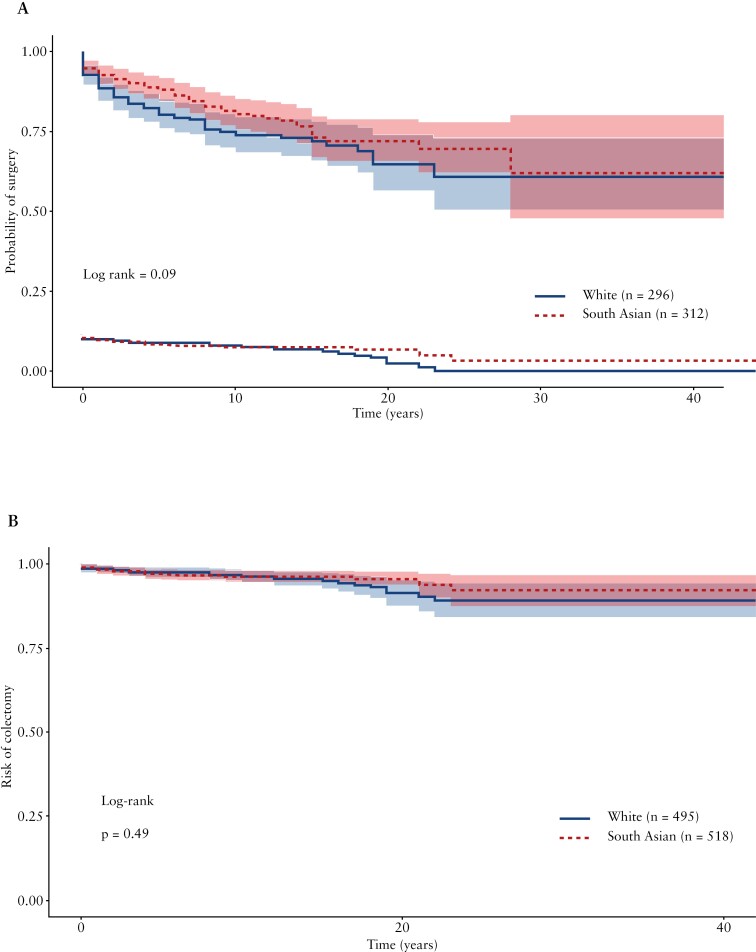
Kaplan-Meier survival analysis in propensity-score matched cohorts: Time to surgery in CD (A), Time to colectomy in UC (B).

No differences in the overall risk of surgery (Figure 2A; hazard ratio [HR] 0.75 [95% CI 0.54-1.05], *p* = 0.09) or time to surgery (Figure 3A; log-rank 0.09) were found between SA and WH. Likewise, there were no differences in the risk of perianal surgery between matched SA and WH with perianal involvement (HR 0.93 [95% CI 0.59-1.47], *p* = 0.76) nor in the time to surgery (Supplementary Figure 3, log-rank 1.00).

The use of biologics has improved IBD prognosis, delaying or avoiding the need to require surgery. Therefore, we also assessed the risk of surgery in a subset of matched patients that were diagnosed in or after the year of approval of the first biologic available in CD (1999) and found no difference in risk of surgery (HR 0.80 [95% CI 0.56-1.15], *p* = 0.23).

#### 3.4.2. Propensity-matched analysis in UC

Twenty-five (4.8%) SA and 26 (5.3%) WH underwent a colectomy in these groups. No demographic or disease extent-related factors were found to be associated with an increased risk of colectomy. Corticosteroids, thiopurines, and biologic use were not associated with any significant difference in risk of having a colectomy (Figure 2B).

No difference in the risk of colectomy (Figure 2B; HR 1.15 [95% CI 0.59-2.22], *p* = 0.69) or time to colectomy (Figure 3B; log-rank *p* = 0.49) was found between the SA and WH cohorts.

No differences in the risk of colectomy were found between WH and SA in those diagnosed after the approval of biologics for UC (2008; HR 1.26 [95% CI 0.58-2.71], p = 0.56).

## 4. Discussion

Non-white ethnic groups have historically been underrepresented in IBD research, and there have been calls for research efforts to be directed toward these groups of patients.^1^ Using the UK IBD-BR, the largest prospective cohort of IBD patients in the UK, this study showed key differences in IBD phenotype between SA and WH IBD, more specifically, in prevalence of IBD subtype, age at diagnosis, smoking history, and sex. Notwithstanding these differences, treatment and surgical management were comparable in SA and WH patients with similar disease phenotypes.

Consistent with previous findings in smaller cohorts,^7,9,13^ UC was the prevalent disease subtype, and a male preponderance was identified in SA. This may be accounted for by ethnic- and sex-related genetic susceptibility^25,26^ but may also stem from higher rates of non-smoking in SA, known to have a causal relationship with UC,^27^ gene-environment interactions, and the microbiome.^28^

SA were younger at diagnosis than WH. In a Canadian pediatric IBD inception cohort study, SA were diagnosed 17 months younger than Caucasians.^29^ However, a cross-sectional study of SA and WH IBD in North West London and a US single-center retrospective study did not identify such differences.^9,13^ These discrepancies could be attributed to their small sample sizes, rendering them insufficiently powered, and the relatively small geographic regions covered. The observed younger age at diagnosis of IBD in SA is consistent with evidence showing that earlier immigration to a country with a high IBD prevalence correlates with an earlier IBD diagnosis.^10,30^ An interaction between genetic factors and environmental exposures relating to dietary and lifestyle changes, pollution, and other elements of the exposome may influence the age of disease onset in the SA migrant population.^31^

The predominance of UC (over CD) and certain phenotypic findings within the SA cohort appear independent of the country of residence. A multicenter, cross-sectional, prospective registry study of nearly 4000 patients across North, East, South, and West India demonstrated UC as the predominant IBD subtype and a male preponderance in both CD and UC.^32^ Studies in India, Nepal, Bangladesh, and Sri Lanka have demonstrated ileocolonic involvement as the prevailing disease location in CD and left-sided disease in UC.^32–35^ However, a lower prevalence of perianal disease in CD (8%,^34^ 2.5%,^33^ 19%^36^ vs 38.5% in this study) and extensive disease in UC (30%^34^ and 35%^33^ vs 41.4%) has been found in patients residing in South Asia.

Previous studies have suggested disparities in the provision of medical therapies in ethnic minority groups.^11^ No significant differences were observed in the medical or surgical approaches to the management of WH and SA with similar disease phenotypes. This is the first study to demonstrate the consistent provision of IBD care in the UK National Health Service. Although prior literature has indicated a higher prevalence of extensive disease and lower rates of colectomy in UC among SA (vs WH), suggesting reduced disease severity,^9^ this study has demonstrated that ethnicity does not influence the time to or risk of colectomy in phenotypically similar cohorts. In the context of CD, comparable surgical outcomes among SA and WH with similar disease phenotypes were observed.

Overall, the main strengths of this study lie in the large number of deeply phenotyped patients included in the IBD-BR dataset, which encompasses specialist and non-specialist centres across the UK, providing a heterogenous real-life study population. Propensity-matched analysis has been used to enhance the validity of the comparisons made in terms of medical and surgical treatments. An average of 97% of the data was complete across the fields used for analysis. We acknowledge the limitations of this study. While it is important to acknowledge the difficulty in defining ethnicity, particularly in view of the increasing admixture of cultures, the use of self-identified ethnicity has been shown to be sufficient to categorise individuals into ethnic groups.^37^ Other ethnic groups could not be included due to low numbers. It is also important to acknowledge that potential ethnic variations in approach to healthcare and research participation may introduce a degree of selection bias. The IBD-BR does not currently collect socioeconomic and migration status data, and therefore an additional constraint lies in the inability of this study to evaluate the impact of deprivation and migration on the observed differences.

This study has demonstrated comparable medical and surgical provision of care in phenotypically matched cohorts. However, we recognise that it is also important to consider the potential influence of ethnicity on the effectiveness of IBD therapies.^38^ Further work will involve exploring the effectiveness of, and adverse events from, IBD therapies in an ethnically diverse patient cohort.

## 5. Conclusion

In the most extensive study of its kind, the UK IBD-BR dataset has been used to reveal notable demographic and phenotypic differences between SA and WH IBD patients. These findings contribute significantly to the limited understanding of the manifestation of IBD in ethnic minorities, a demographic that has been underrepresented in previous IBD studies and may provide further opportunities to personalise healthcare.^6^ Furthermore, these variations underscore the critical need for research studies to include participants from diverse ethnic backgrounds to reflect the real-world population to strengthen the generalisability of research findings. Another key point to emerge from this study is that the provision of medical and surgical care in phenotypically comparable cohorts is similar in the UK, indicating that the current algorithms used to manage patients are being appropriately applied to ethnically diverse patient cohorts.

## Supplementary Material

jjae186_suppl_Supplementary_Tables_S1-S7_Figures_S1-S3

## Data Availability

Patient-level data underlying this study are available to researchers subject to the access processes of the UK IBD BioResource, detailed further at https://www.ibdbioresource.nihr.ac.uk/index.php/resources.
