# Supplementary material for: Diverse Phenotypes, Consistent Treatment: A Study of 30 997 South Asian and White Inflammatory Bowel Disease Patients Using the UK Inflammatory Bowel Disease BioResource
Source: J Crohns Colitis. 2024 Dec 7;19(1):jjae186. doi: 10.1093/ecco-jcc/jjae186 (PMC11737890; doi:10.1093/ecco-jcc/jjae186)
Supplement: jjae186_suppl_Supplementary_Tables_S1-S7_Figures_S1-S3 [file jjae186_suppl_supplementary_tables_s1-s7_figures_s1-s3.docx]

**Supplementary material: text**

We performed a comparative analysis of the degree of similarity or difference of key clinical variables between SA and WH patients, including all relevant patients in the extracted patient cohort.

***Unmatched analysis of medication use: CD (Supplementary Table 5)***

A younger age at diagnosis was associated with an increased risk of corticosteroid [OR 0.88 (95% CI 0.84-0.92), p<0.001], thiopurine [OR 0.67 ( 95% CI 0.64-0.70), p<0.001], and anti-TNF use [OR 0.61 (95% CI 0.58-0.64), p<0.001]. Older age at diagnosis was associated with an increased vedolizumab use [OR 1.41 (95% CI 1.23-1.61), p<0.001]. Female sex was associated with increased corticosteroid [OR 1.14 (95% CI 1.06-1.23), p<0.001] and vedolizumab [OR 1.64 (95% CI 1.21-2.24), p=0.002] use. A smoking history at the time of diagnosis was associated with corticosteroid [ex-smoker, OR 1.20 (95% CI 1.10-1.30), p<0.001; current smoker, OR 1.36 (95% CI 1.22-1.51), p<0.001] but not thiopurine or biologic use. Corticosteroid [OR 1.21 (95% CI 1.11-1.33), p<0.001], thiopurine [OR 1.49 (95% CI 1.34-1.65), p<0.001] and anti-TNF [OR 1.77 (1.59-1.96), p<0.001] use was associated with ileocolonic (relative to ileal) disease, but this was not seen with vedolizumab. Anti-TNF use was also associated with colonic [OR 1.40 (95% CI 1.25-1.56), p<0.001] and upper GI [OR 1.56 (95% CI 1.04-2.35), p=0.03] disease. Stricturing disease was associated with corticosteroid use [] but this was not seen in those with penetrating disease or perianal involvement. Penetrating, stricturing, and perianal disease were associated with thiopurine and anti-TNF but not vedolizumab use.

***Unmatched analysis of medication use: UC (Supplementary Table 6)***

A younger age at diagnosis was associated with corticosteroid [OR 0.79 (95% CI 0.76-0.82), p<0.001], thiopurine [OR 0.67 (95% CI 0.65-0.70), p<0.001] and anti-TNF [OR 0.63 (95% CI 0.59-0.66), p<0.001] use. Female sex was associated with reduced thiopurine [OR 0.81 (95% CI 0.75-0.87), p<0.001] use. Smoking at the time of diagnosis was associated with a reduced thiopurine [OR 0.72 (95% CI 0.61-0.84), p<0.001] and biologic use [anti-TNFs, OR 0.74 (0.59-0.93), p=0.009; vedolizumab, OR 0.48 (95% CI 0.28-0.78), p=0.005]. Extensive disease was associated with corticosteroid [OR 2.59 (95% CI 2.32-2.89), p<0.001], thiopurine [OR 3.92 (95% CI 3.48-4.42), p<0.001], anti-TNF [OR 4.83 (95% CI 4.02-5.85), p<0.001] and vedolizumab [OR 4.75 (95% CI 3.21-7.28), p<0.001] use.

***Unmatched analysis of surgery: CD (Supplementary Table 7)***

Female sex [HR 1.18 (95% CI 1.11-1.24), p<0.001], a smoking history at the time of diagnosis [current smoker, HR 1.13 (95% CI 1.04-1.23), p=0.005; ex-smoker, HR 1.11 (1.03-1.18), p=0.004], increased disease duration (see **Supplementary Table 7**) and severe disease phenotype [penetrating, HR 3.06 (95% CI 2.81-3.33), p<0.001; stricturing, HR 2.70 (95% CI 2.51-2.90), p<0.001] were associated with an increased risk of surgery. Ileal disease was also associated with an increased risk of surgery relative to colonic, ileocolonic and exclusive upper GI involvement. Perianal involvement was not associated with a difference in risk of surgery (intestinal resection) [HR 0.99 (95% CI 0.93-1.06), p=0.88].

In those with perianal involvement, an increased risk of perianal intervention was associated with a history of smoking at the time of diagnosis [HR 1.25 (95% CI 1.06-1.47), p=0.008], a shorter disease duration [relative to less than five years: 5-9 years, HR 0.58 (95% CI 0.42-0.82), p=0.002; 10-14 years, HR 0.50 (95% CI 0.36-0.71), p<0.001; 15-19 years, HR 0.30 (95% CI 0.21-0.42), p<0.001; 20 years or more, HR 0.18 (95% CI 0.13-0.26), p<0.001], colonic and ileocolonic disease [colonic, HR 2.13 (95% CI 1.78-2.55), p<0.001; ileocolonic, HR 1.74 (95% CI 1.46-2.07), p<0.001], and penetrating disease [HR 1.27 (95% CI 1.09-1.47), p=0.002]. Female sex was associated with a lower risk of perianal intervention [HR 0.76 (95% CI 0.68-0.86), p<0.001].

***Unmatched analysis of surgery: UC (Supplementary Table 7)***

Shorter disease duration was associated with an increased risk of colectomy [relative to less than 5 years: 5-9 years, HR 0.52 (95% CI 0.27-0.99), p=0.47; 10-14 years, HR 0.23 (95% CI 0.12-0.44), p<0.001; 15-19 years, HR 0.13 (95% CI 0.07-0.25), p<0.001; 20 years or more, HR 0.04 (95% CI 0.02-0.09), p<0.001]. Sex, smoking history at diagnosis, and disease extent-associations with risk of colectomy were not identified.

**Supplementary material: figures and tables**


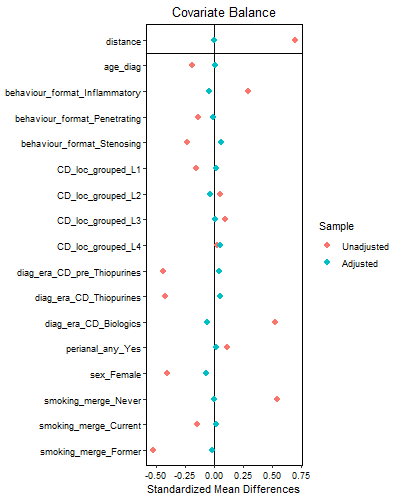


Unadjusted

Adjusted

**Supplementary Figure 1**

**SMD in baseline covariates between unmatched and propensity score-matched patients in CD**

**A) Corticosteroids; B) Thiopurines; C) Anti-TNFs; D) Vedolizumab; E) Surgery**


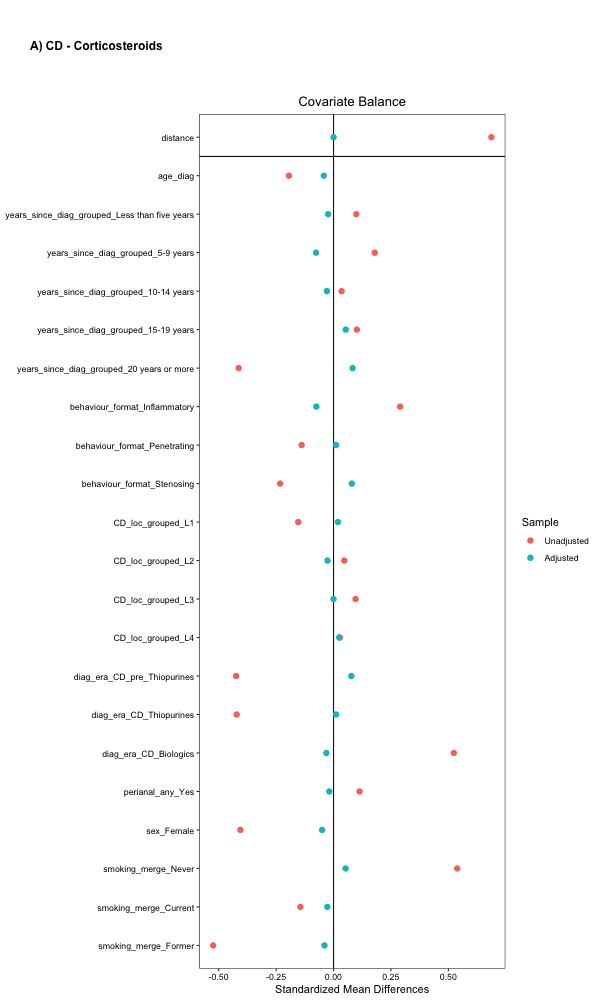

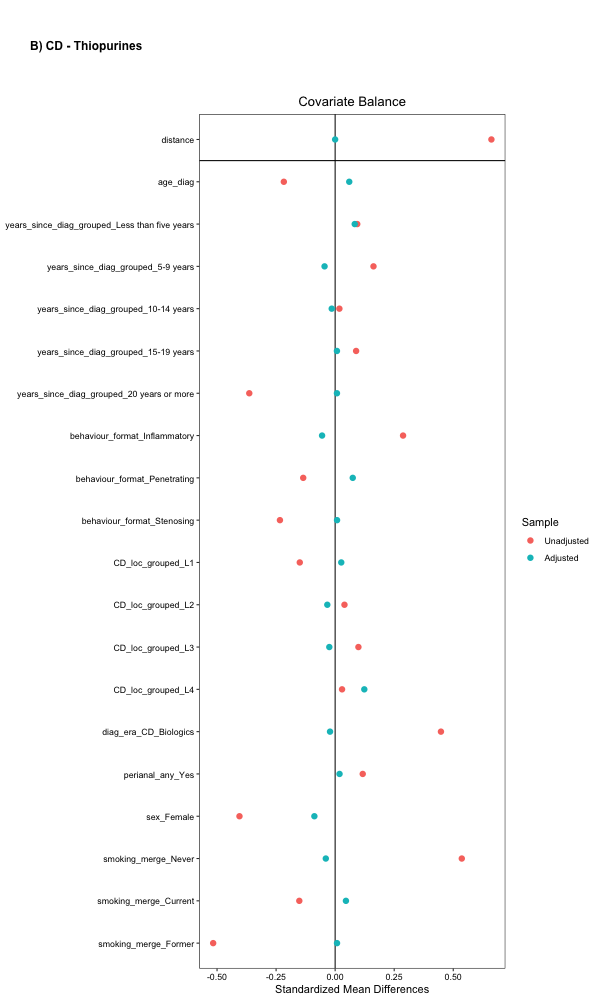

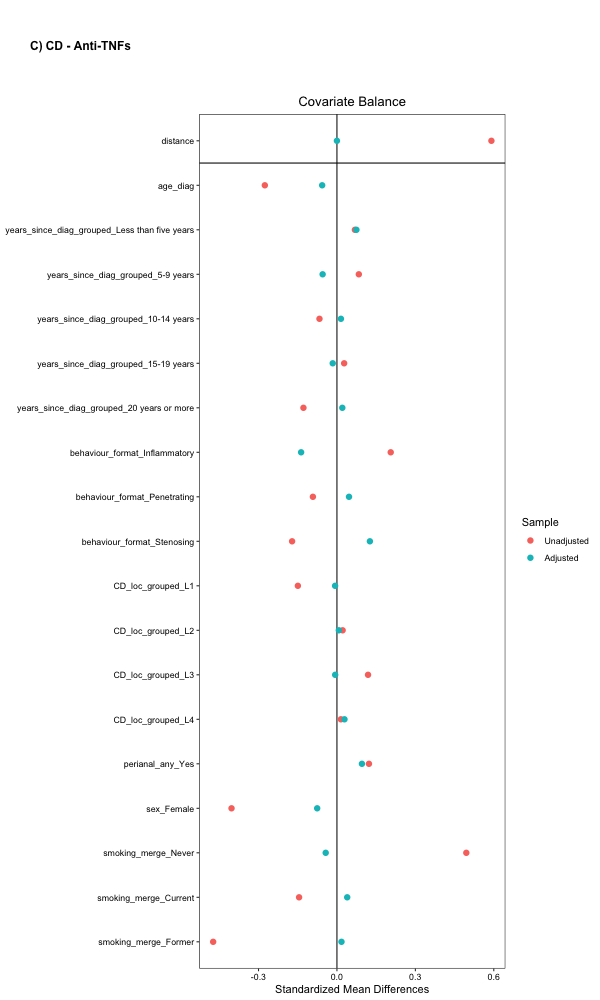

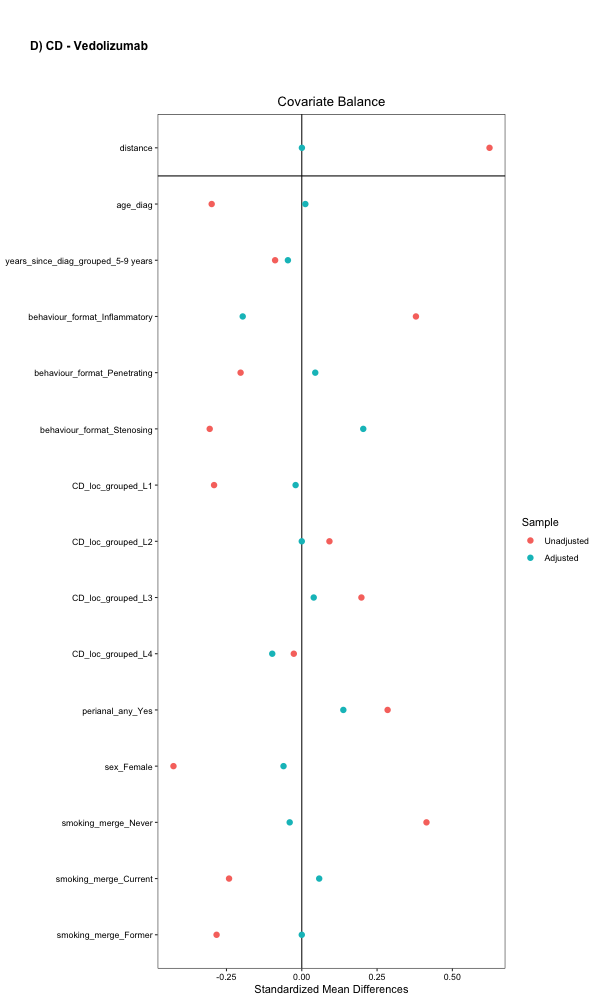

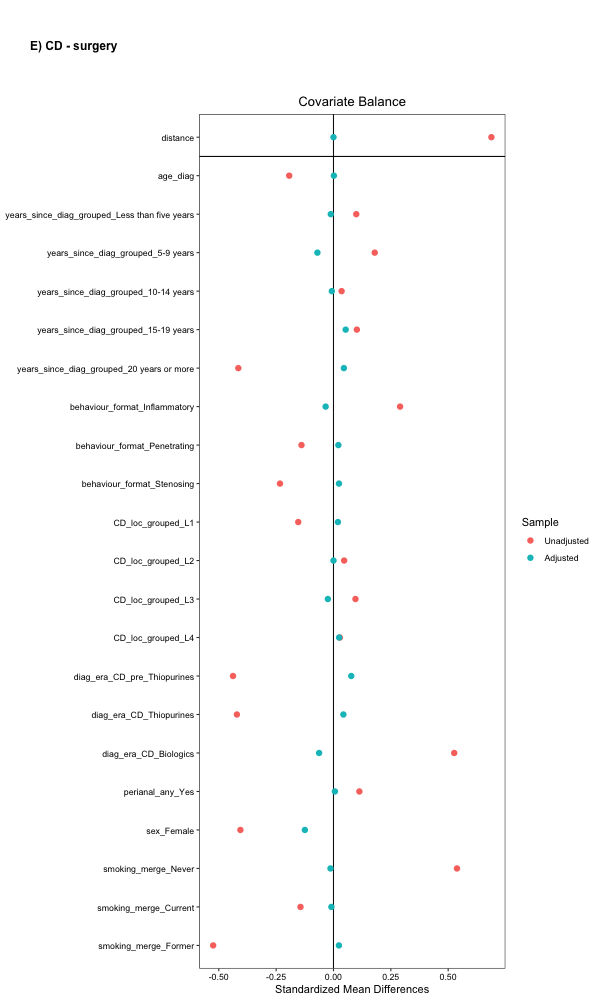


**Supplementary Figure 2**

**SMD in baseline covariates between unmatched and propensity score-matched patients in UC**

**A) Aminosalicylates and corticosteroids; B) Thiopurines; C) Anti-TNFs; D) Vedolizumab; E) Colectomy**


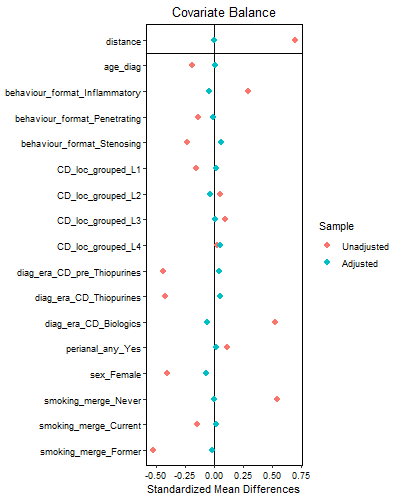


Unadjusted

Adjusted


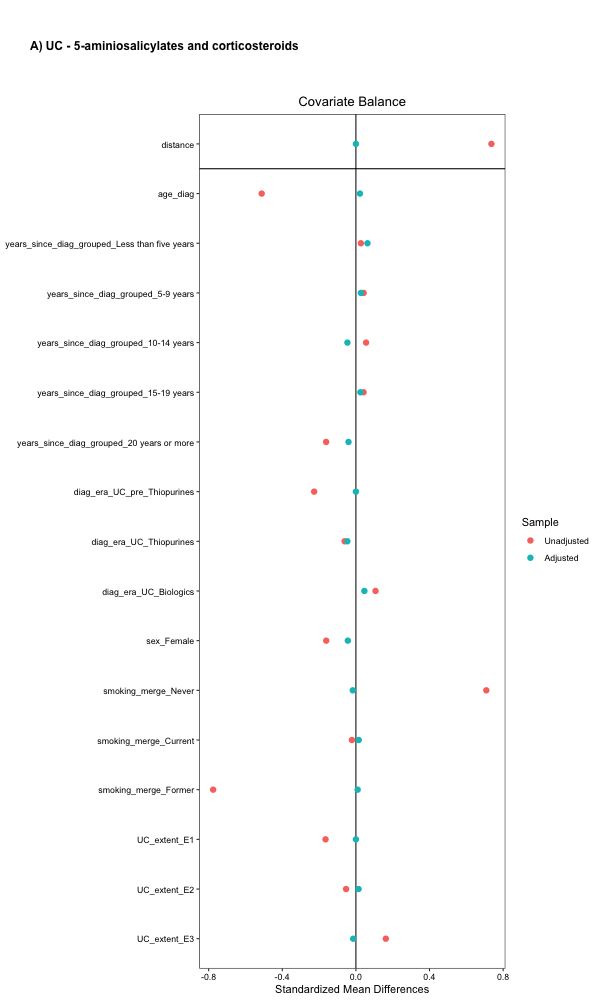

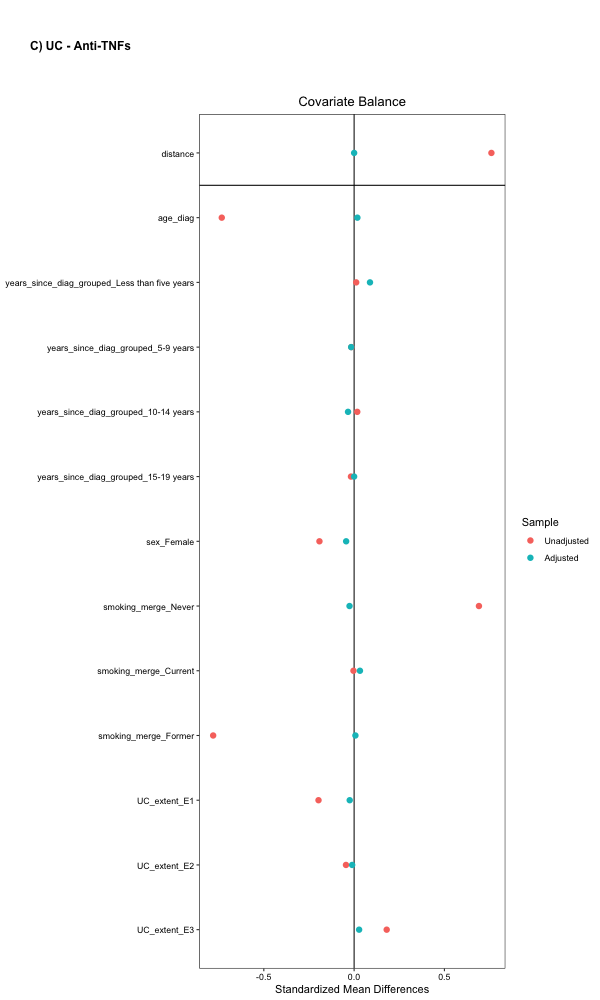

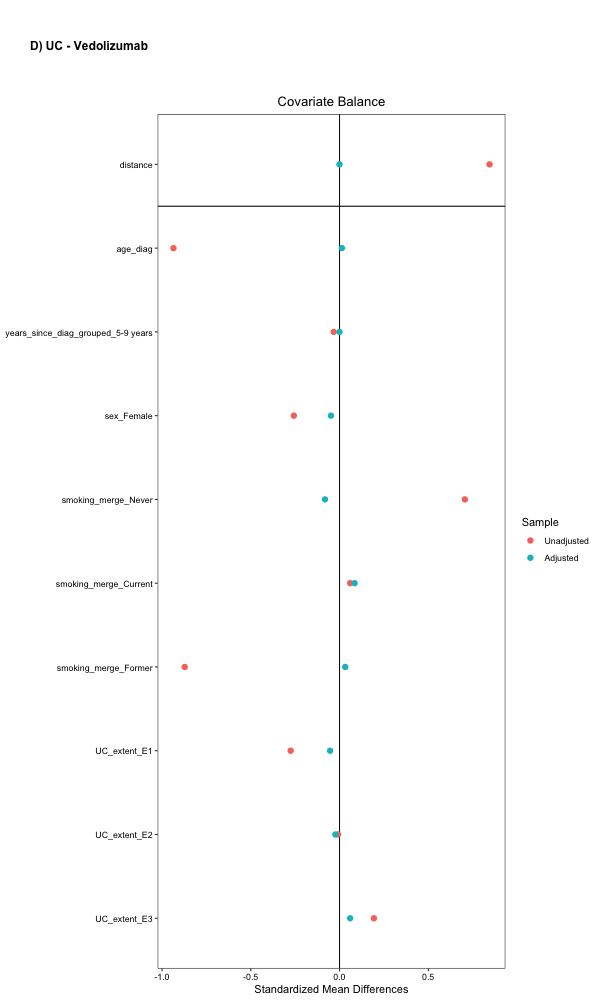

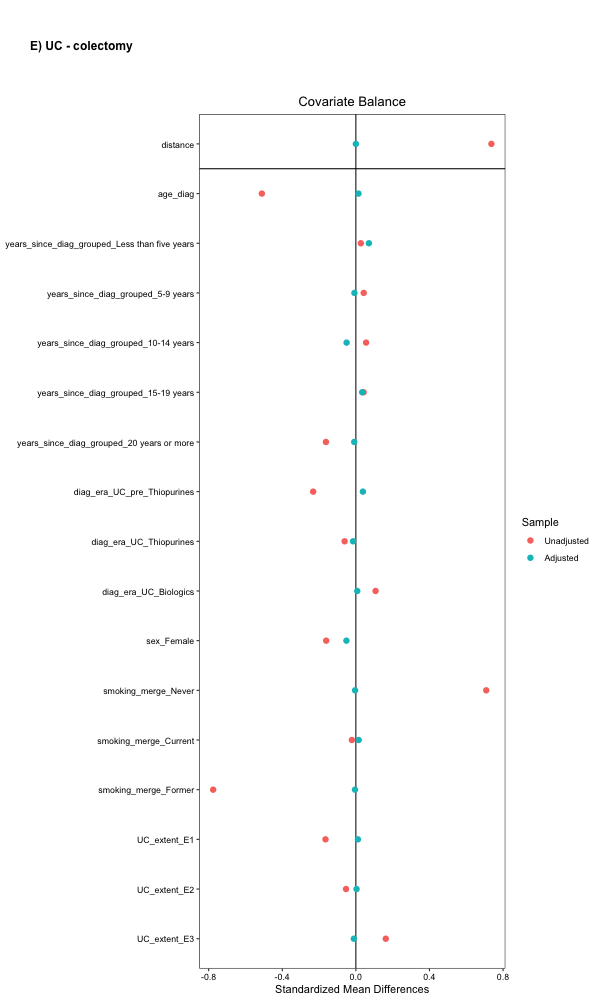

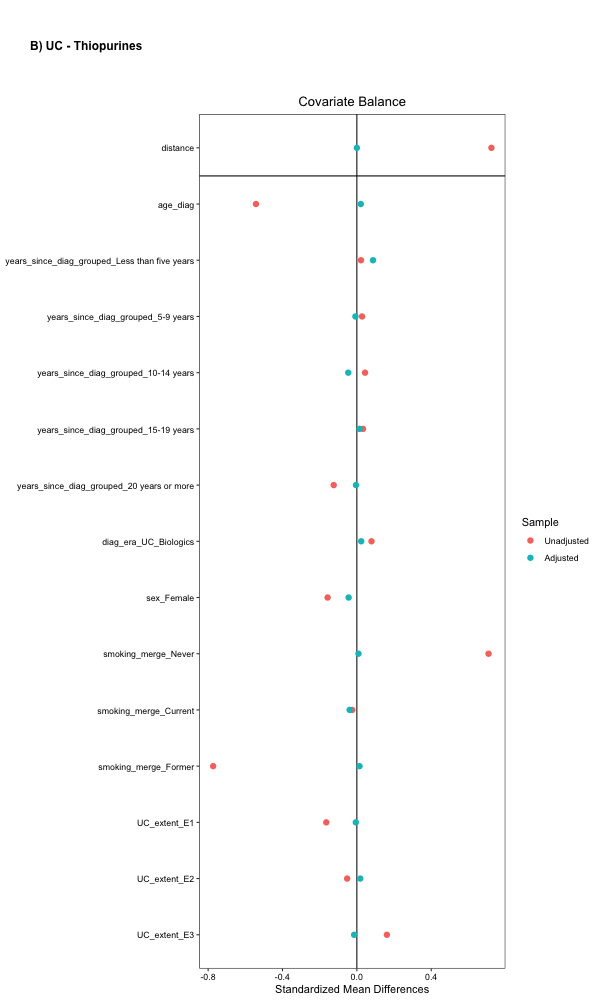

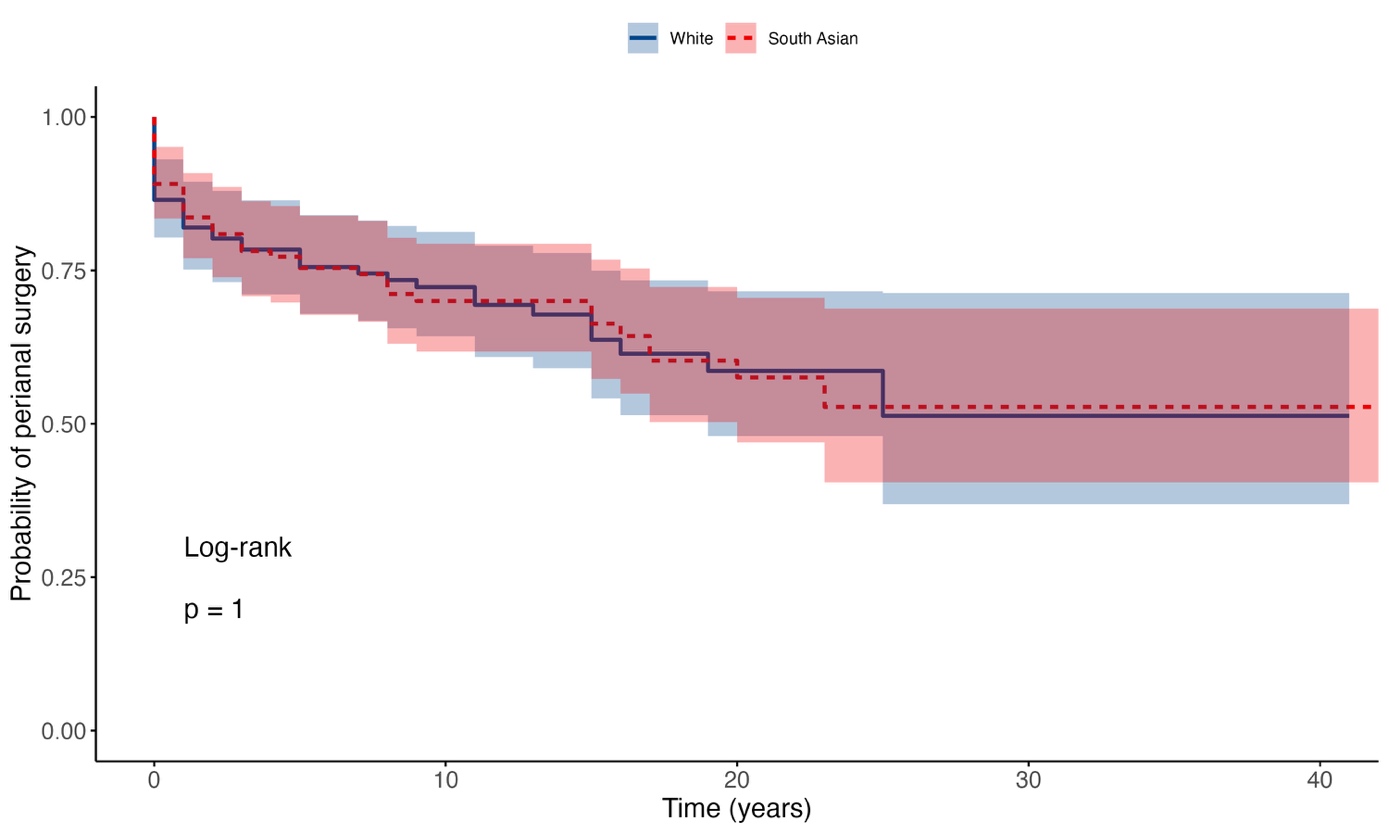


**Supplementary Figure 3**

**Kaplan-Meier survival analysis in propensity-matched cohort in CD in subset of patients with perianal disease**


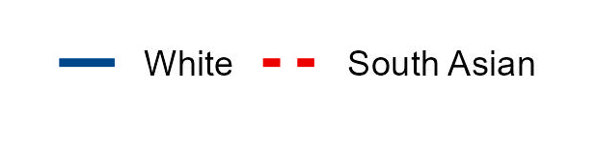

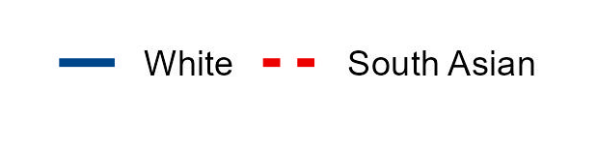


White (n=111)

South Asian (n=110)

Log rank = 1.00

| **Supplementary Table 1 Demographic and phenotypic characteristics of Black, South Asian and White patients with CD** | | | | |  |
| --- | --- | --- | --- | --- | --- |
|  | **White**  **N = 15507** | **South Asian**  **N = 437** | **Black**  **N = 78** | **p** | |
| **Sex, N (%)** |  |  |  | <0.001*** | |
| Female | 8477 (54.7) | 160 (36.6) | 35 (44.9) |  | |
| Male | 7030 (45.3) | 277 (63.4) | 43 (55.1) |  | |
| **Age (years) at diagnosis, Median (IQR)** | 26 (19-39) | 24 (17-36) | 22 (14-32) | <0.001*** | |
| **Smoking status, N (%)** |  |  |  | <0.001*** | |
| Never smoked | 6715 (46.1) | 299 (72.7) | 40 (54.8) |  | |
| Ex-smoker | 5385 (36.9) | 68 (16.5) | 11 (15.1) |  | |
| Current smoker | 2475 (17.0) | 44 (10.7) | 22 (30.1) |  | |
| **Disease location, N (%)** |  |  |  | <0.001*** | |
| Ileal | 5765 (38.4) | 124 (30.3) | 20 (27.0) |  | |
| Colonic | 3972 (26.4) | 121 (29.6) | 30 (40.5) |  | |
| Ileo-colonic | 5108 (34.0) | 158 (38.6) | 20 (27.0) |  | |
| Isolated upper GI | 187 (1.2) | 6 (1.5) | 4 (5.4) |  | |
| **Disease behaviour, N (%)** |  |  |  | <0.001*** | |
| Non-stricturing, non-penetrating | 8975 (61.7) | 289 (73.9) | 40 (54.1) |  | |
| Stricturing | 3722 (25.6) | 66 (16.9) | 24 (32.4) |  | |
| Penetrating | 1841 (12.7) | 36 (9.2) | 10 (13.5) |  | |
| Perianal involvement | 4677 (32.2) | 153 (38.5) | 39 (54.9) |  | |
| * p <0.05, ** p <0.001, *** p <0.001 | | | | | |

| Supplementary Table 2 Demographic and phenotypic characteristics of Black, South Asian and White patients with UC | | | | |  |
| --- | --- | --- | --- | --- | --- |
|  | **White**  **N = 14531** | **South Asian**  **N = 702** | **Black**  **N = 73** | **p** | |
| Sex, N (%) |  |  |  | <0.001*** | |
| Female | 7131 (49.7) | 289 (41.2) | 42 (57.5) |  | |
| Male | 7220 (50.3) | 413 (58.8) | 31 (42.5) |  | |
| Age (years) at diagnosis, Median (IQR) | 35 (25-48) | 29 (22-38) | 34 (24-42) | <0.001*** | |
| Smoking status, N (%) |  |  |  | <0.001*** | |
| Never smoked | 6359 (46.8) | 510 (77.5) | 41 (58.6) |  | |
| Ex-smoker | 6339 (46.7) | 113 (17.2) | 19 (27.1) |  | |
| Current smoker | 876 (6.5) | 35 (5.3) | 10 (14.3) |  | |
| Disease extent, N (%) |  |  |  | 0.001** | |
| Proctitis | 2184 (17.2) | 72 (12.1) | 12 (18.5) |  | |
| Left-sided | 6178 (48.7) | 274 (46.2) | 31 (47.7) |  | |
| Extensive | 4321 (34.1) | 247 (41.7) | 22 (33.8) |  | |
| ** p <0.05, ** p <0.001,* *** *p* <0*.001* | | | | | |

| Supplementary Table 3 Demographic and phenotypic characteristics of White vs Bangladeshi/Indian/Pakistani patients with CD | | | | | | | | | | | | |
| --- | --- | --- | --- | --- | --- | --- | --- | --- | --- | --- | --- | --- |
|  | **White** | **Bangladeshi** | **p** | **p_adj_** | **White** | **Indian** | **p** | **p_adj_** | **White** | **Pakistani** | **p** | **p_adj_** |
| Total N (%) | 15507 (99.4) | 88 (0.6) |  |  | 15507 (98.5) | 242 (1.5) |  |  | 15507 (99.3) | 107 (0.7) |  |  |
| Sex, N (%) |  |  | <0.001*** | _ |  |  | <0.001*** | _ |  |  | <0.001*** | _ |
| Female | 8477 (54.7) | 25 (28.4) |  |  | 8477 (54.7) | 98 (40.5) |  |  | 8477 (54.7) | 37 (34.6) |  |  |
| Male | 7030 (45.3) | 63 (71.6) |  |  | 7030 (45.3) | 144 (59.5) |  |  | 7030 (45.3) | 70 (65.4) |  |  |
| Age (years) at diagnosis, Median (IQR) | 26 (19 - 39) | 23 (16 - 31) | <0.001*** | _ | 26 (19 - 39) | 28 (18 - 40) | 0.93 | _ | 26 (19 - 39) | 20 (15 - 32) | <0.001*** | _ |
| Smoking status, N (%) |  |  | 0.02* |  |  |  | <0.001*** |  |  |  | <0.001 |  |
| Never smoked | 6715 (46.1) | 45 (59.2) |  | 0.07 | 6715 (46.1) | 181 (77.7) |  | <0.001*** | 6715 (46.1) | 73 (71.6) |  | <0.001*** |
| Ex-smoker | 2475 (17.0) | 15 (19.7) |  | 1.00 | 2475 (17.0) | 17 (7.3) |  | <0.001*** | 2475 (17.0) | 12 (11.8) |  | 0.32 |
| Current smoker | 5385 (36.9) | 16 (21.1) |  | 0.03* | 5385 (36.9) | 35 (15.0) |  | <0.001*** | 5385 (36.9) | 17 (16.7) |  | <0.001*** |
| Disease location, N (%) |  |  | 0.001** |  |  |  | 0.07 |  |  |  | 0.30 |  |
| Ileal | 5765 (38.4) | 22 (26.5) |  | 0.11 | 5765 (38.4) | 71 (31.3) |  |  | 5765 (38.4) | 31 (31.3) |  |  |
| Colonic | 3972 (26.4) | 15 (18.1) |  | 0.22 | 3972 (26.4) | 72 (31.7) |  |  | 3972 (26.4) | 34 (34.3) |  |  |
| Ileo-colonic | 5108 (34.0) | 46 (55.4) |  | <0.001*** | 5108 (34.0) | 79 (34.8) |  |  | 5108 (34.0) | 33 (33.3) |  |  |
| Isolated upper GI | 187 (1.2) | 0 (0.0) |  | 0.61 | 187 (1.2) | 5 (2.2) |  |  | 187 (1.2) | 1 (1.0) |  |  |
| Disease behaviour, N (%) |  |  | 0.35 |  |  |  | <0.001*** |  |  |  | 0.17 |  |
| Non-stricturing, non-penetrating | 8975 (61.7) | 55 (67.9) |  |  | 8975 (61.7) | 167 (77.3) |  | <0.001*** | 8975 (61.7) | 67 (71.3) |  |  |
| Stricturing | 1841 (12.7) | 11 (13.6) |  |  | 1841 (12.7) | 16 (7.4) |  | 0.04* | 1841 (12.7) | 9 (9.6) |  |  |
| Penetrating | 3722 (25.6) | 15 (18.5) |  |  | 3722 (25.6) | 33 (15.3) |  | 0.001*** | 3722 (25.6) | 18 (19.1) |  |  |
| Perianal involvement | 4677 (32.2) | 36 (42.9) | 0.049* |  | 4677 (32.2) | 79 (36.2) | 0.23 |  | 4677 (32.2) | 38 (40.0) | 0.13 |  |
| ** p <0.05, ** p <0.001,* *** *p* <0*.001* | | | | | | | | | | | | |

| Supplementary Table 4 Demographic and phenotypic characteristics of White vs Bangladeshi/Indian/Pakistani patients with UC | | | | | | | | | | | | | |
| --- | --- | --- | --- | --- | --- | --- | --- | --- | --- | --- | --- | --- | --- |
|  | **White** | **Bangladeshi** | **p** | **p_adj_** | **White** | **Indian** | **p** | **p_adj_** | **White** | **Pakistani** | **p** | **p_adj_** |  |
| Total, N (%) | 14351 (99.6) | 55 (0.4) |  |  | 14351 (96.6) | 505 (3.4) |  |  | 14351 (99.0) | 142 (1.0) |  |  |  |
| Sex, N (%) |  |  | 0.12 | _ |  |  |  |  |  |  |  |  |  |
| Female | 7131 (49.7) | 21 (38.2) |  |  | 7131 (49.7) | 204 (40.4) | <0.001*** | _ | 7131 (49.7) | 64 (45.1) | 0.31 | _ |  |
| Male | 7220 (50.3) | 34 (61.8) |  |  | 7220 (50.3) | 301 (59.6) |  |  | 7220 (50.3) | 78 (54.9) |  |  |  |
| Age (years) at diagnosis, Median (IQR) | 35 (25 - 48) | 27 (20 - 36) | <0.001*** | _ | 35 (25 - 48) | 30 (22 - 40) | <0.001*** | _ | 35 (25 - 48) | 27 (20 - 33) | <0.001*** | _ |  |
| Smoking status, N (%) |  |  | <0.001*** |  |  |  | <0.001*** |  |  |  | <0.001*** |  |  |
| Never smoked | 6359 (46.8) | 38 (74.5) |  | <0.001*** | 6359 (46.8) | 361 (75.8) |  | <0.001*** | 6359 (46.8) | 111 (84.7) |  | <0.001*** |  |
| Ex-smoker | 876 (6.5) | 4 (7.8) |  | 1.00 | 876 (6.5) | 25 (5.3) |  | 0.59 | 876 (6.5) | 6 (4.6) |  | 0.77 |  |
| Current smoker | 6339 (46.7) | 9 (17.6) |  | <0.001*** | 6339 (46.7) | 90 (18.9) |  | <0.001*** | 6339 (46.7) | 14 (10.7) |  | <0.001*** |  |
| Disease extent, N (%) |  |  | 0.15 |  |  |  | 0.004** |  |  |  | 0.007** |  |  |
| Proctitis | 2184 (17.2) | 9 (18.0) |  |  | 2184 (17.2) | 48 (11.5) |  | 0.01* | 2184 (17.2) | 15 (12.0) |  | 0.25 |  |
| Left-sided | 6178 (48.7) | 18 (36.0) |  |  | 6178 (48.7) | 205 (49.0) |  | 1.00 | 6178 (48.7) | 51 (40.8) |  | 0.23 |  |
| Extensive | 4321 (34.1) | 23 (46.0) |  |  | 4321 (34.1) | 165 (39.5) |  | 0.07 | 4321 (34.1) | 59 (47.2) |  | 0.01* |  |
| ** p <0.05, ** p <0.001,* *** *p* <0*.001* | | | | | | | | | | | | | |

| **Supplementary Table 5 Adjusted OR for medication use in White (WH) vs South Asian (SA) IBD patients in unmatched cohorts in CD**  **WH – referent group** | | | | | | | | |
| --- | --- | --- | --- | --- | --- | --- | --- | --- |
| **N (%)**  WH  SA | **Corticosteroids**  5489 (35.6)  262 (60.2) | | **Thiopurines**  10923 (73.9)  321 (74.3) | | **Anti-TNFs**  6893 (59.1)  241 (61.5) | | **Vedolizumab**  227 (6.2)  11 (8.3) | |
|  | **OR (95% CI)** | **p** | **OR (95% CI)** | **p** | **OR (95% CI)** | **p** | **OR (95% CI)** | **p** |
| **Age at diagnosis** | 0.88 (0.84-0.92) | <0.001*** | 0.67 (0.64-0.70) | <0.001*** | 0.61 (0.58-0.64) | <0.001*** | 1.41 (1.23-1.61) | <0.001*** |
| **Sex** |  |  |  |  |  |  |  |  |
| Male | _ | _ | _ | _ | _ | _ | _ | _ |
| Female | 1.14 (1.06-1.23) | <0.001*** | 0.99 (0.91-1.08) | 0.85 | 0.96 (0.88-1.04) | 0.33 | 1.64 (1.21-2.24) | 0.002** |
| **Ethnicity** |  |  |  |  |  |  |  |  |
| White | _ | _ | _ | _ | _ | _ | _ | _ |
| South Asian | 0.90 (0.72-1.14) | 0.39 | 1.05 (0.81-1.38) | 0.72 | 0.86 (0.67-1.11) | 0.25 | 1.73 (0.79-3.37) | 0.14 |
| **Smoking status** |  |  |  |  |  |  |  |  |
| Never smoker | _ | _ | _ | _ | _ | _ | _ | _ |
| Ex-smoker | 1.20 (1.10-1.30) | <0.001*** | 0.97 (0.88-1.07) | 0.59 | 0.96 (0.87-1.06) | 0.40 | 1.12 (0.80-1.56) | 0.52 |
| Current smoker | 1.36 (1.22-1.51) | <0.001*** | 1.03 (0.92-1.17) | 0.58 | 1.00 (0.89-1.13) | 1.00 | 0.79 (0.50-1.20) | 0.28 |
| **Disease duration (years)** |  |  |  |  |  |  |  |  |
| <5 | _ | _ | _ | _ | _ | _ | _ | _ |
| 5-9 | 0.94 (0.78-1.13) | 0.51 | 2.04 (1.69-2.45) | <0.001*** | 0.66 (0.55-0.80) | <0.001*** | 0.45 (0.33-0.62) | <0.001*** |
| 10-14 | 0.95 (0.78-1.15) | 0.59 | 3.71 (3.06-4.50) | <0.001*** | 0.81 (0.67-0.99) | 0.04* | _ | _ |
| 15-19 | 1.04 (0.85-1.27) | 0.68 | 3.34 (2.73-4.09) | <0.001*** | 0.63 (0.51-0.77) | <0.001*** | _ | _ |
| ≥20 | 0.96 (0.79-1.15) | 0.64 | 2.73 (2.26-3.29) | <0.001*** | 0.56 (0.45-0.69) | <0.001*** | _ | _ |
| **Disease location** |  |  |  |  |  |  |  |  |
| Ileal | _ | _ | _ | _ | _ | _ | _ | _ |
| Colonic | 1.04 (0.94-1.14) | 0.47 | 1.00 (0.90-1.11) | 0.97 | 1.40 (1.25-1.56) | <0.001*** | 0.96 (0.66-1.39) | 0.84 |
| Ileocolonic | 1.21 (1.11-1.33) | <0.001*** | 1.49 (1.34-1.65) | <0.001*** | 1.77 (1.59-1.96) | <0.001*** | 0.90 (0.61-1.30) | 0.57 |
| Exclusive upper GI | 0.79 (0.57-1.10) | 0.16 | 1.12 (0.77-1.68) | 0.56 | 1.56 (1.04-2.35) | 0.03* | 0.95 (0.15-3.25) | 0.95 |
| **Disease behaviour** |  |  |  |  |  |  |  |  |
| Inflammatory | _ | _ | _ | _ | _ | _ | _ | _ |
| Penetrating | 0.93 (0.83-1.04) | 0.20 | 1.19 (1.04-1.37) | 0.01* | 1.34 (1.16-1.55) | <0.001*** | 0.81 (0.44-1.40) | 0.49 |
| Stricturing | 1.14 (1.04-1.24) | 0.006** | 1.50 (1.34-1.67) | <0.001*** | 1.66 (1.48-1.85) | <0.001*** | 1.16 (0.78-1.69) | 0.44 |
| **Perianal involvement** | 0.98 (0.90-1.07) | 0.66 | 1.36 (1.23-1.50) | <0.001*** | 2.28 (2.06-2.52) | <0.001*** | 1.19 (0.82-1.71) | 0.34 |
| * p <0.05, ** p <0.001, *** p <0.001  Adjusted for age at diagnosis, sex, ethnicity, smoking status, disease duration, disease location, disease behaviour and perianal disease | | | | | | | | |

| **Supplementary Table 6 Adjusted OR for medication use in White (WH) vs South Asian (SA) IBD patients in unmatched cohorts in UC**  **WH – referent group** | | | | | | | | | | |
| --- | --- | --- | --- | --- | --- | --- | --- | --- | --- | --- |
| **N (%)**  WH  SA | **Aminosalicylates**  11811 (82.5)  586 (83.7) | | **Corticosteroids**  8747 (61.1)  447 (63.9) | | **Thiopurines**  6384 (46.2)  375 (54.0) | | **Anti-TNFs**  2489 (28.6)  165 (35.6) | | **Vedolizumab**  517 (12.3)  30 (13.7) | |
|  | **OR (95% CI)** | **p** | **OR (95% CI)** | **p** | **OR (95% CI)** | **p** | **OR (95% CI)** | **p** | **OR (95% CI)** | **p** |
| **Age at diagnosis** | 1.02 (0.97-1.08) | 0.36 | 0.79 (0.76-0.82) | <0.001*** | 0.67 (0.65-0.70) | <0.001*** | 0.63 (0.59-0.66) | <0.001*** | 0.93 (0.84-1.02) | 0.13 |
| **Sex** |  |  |  |  |  |  |  |  |  |  |
| Male | _ | _ | _ | _ | _ | _ | _ | _ | _ | _ |
| Female | 1.09 (0.99-1.20) | 0.07 | 0.93 (0.86-1.00) | 0.06* | 0.81 (0.75-0.87) | <0.001*** | 0.91 (0.82-1.01) | 0.07 | 0.82 (0.66-1.00) | 0.50 |
| **Ethnicity** |  |  |  |  |  |  |  |  |  |  |
| White | _ | _ | _ | _ | _ | _ | _ | _ | _ | _ |
| South Asian | 1.07 (0.85-1.36) | 0.57 | 0.95 (0.80-1.15) | 0.62 | 1.09 (0.91-1.31) | 0.33 | 1.08 (0.86-1.36) | 0.50 | 0.88 (0.53-1.37) | 0.58 |
| **Smoking status** |  |  |  |  |  |  |  |  |  |  |
| Never smoker | _ | _ | _ | _ | _ | _ | _ | _ | _ | _ |
| Ex-smoker | 1.07 (0.97-1.19) | 0.20 | 1.11 (1.02-1.20) | 0.01* | 1.12 (1.03-1.21) | 0.008** | 1.35 (1.20-1.51) | <0.001*** | 0.88 (0.70-1.10) | 0.25 |
| Current smoker | 0.88 (0.73-1.08) | 0.21 | 0.98 (0.84-1.14) | 0.77 | 0.72 (0.61-0.84) | <0.001*** | 0.74 (0.59-0.93) | 0.009** | 0.48 (0.28-0.78) | 0.005** |
| **Disease duration (years)** |  |  |  |  |  |  |  |  |  |  |
| <5 | _ | _ | _ | _ | _ | _ | _ | _ | _ | _ |
| 5-9 | 1.41 (1.14-1.74) | 0.002** | 0.80 (0.67-0.96) | 0.02* | 1.88 (1.55-2.28) | <0.001*** | 0.62 (0.52-0.75) | <0.001*** | 0.57 (0.45-0.73, p<0.001) | <0.001*** |
| 10-14 | 1.51 (1.21-1.87) | <0.001*** | 0.89 (0.74-1.07) | 0.23 | 3.03 (2.49-3.70) | <0.001*** | 0.56 (0.46-0.68) | <0.001*** | _ | _ |
| 15-19 | 1.43 (1.14-1.80) | 0.002** | 0.90 (0.74-1.09) | 0.30 | 3.04 (2.48-3.74) | <0.001*** | 0.44 (0.34-0.58) | <0.001*** | _ | _ |
| ≥20 | 1.33 (1.07-1.64) | 0.01* | 0.72 (0.60-0.87) | <0.001*** | 2.05 (1.69-2.50) | <0.001*** | _ | _ | _ | _ |
| **Disease extent** |  |  |  |  |  |  |  |  |  |  |
| Proctitis | _ | _ | _ | _ | _ | _ | _ | _ | _ | _ |
| Left-sided | 0.89 (0.77-1.03) | 0.11 | 2.25 (2.04-2.49) | <0.001*** | 2.65 (2.37-2.98) | <0.001*** | 3.67 (3.07-4.41) | <0.001*** | 3.79 (2.59-5.75) | <0.001*** |
| Extensive | 0.73 (0.63-0.84) | <0.001*** | 2.59 (2.32-2.89) | <0.001*** | 3.92 (3.48-4.42) | <0.001*** | 4.83 (4.02-5.85) | <0.001*** | 4.75 (3.21-7.28) | <0.001*** |
| * p <0.05, ** p <0.001, *** p <0.001  Adjusted for age at diagnosis, sex, ethnicity, smoking status, disease duration and disease extent | | | | | | | | | | |

| **Supplementary Table 7 Adjusted HR for surgery (CD) / colectomy (UC) White (WH) vs South Asian (SA) IBD patients in unmatched cohorts**  **WH – referent group** | | | | | | | |
| --- | --- | --- | --- | --- | --- | --- | --- |
|  | **CD** | |  | | | **UC** | |
|  | **HR (95% CI)** | **p** | |  | **HR (95% CI)** | | **p** |
| **Age at diagnosis** | 1.01 (0.97-1.05) | 0.69 | |  | 1.08 (0.98-1.20) | | 0.12 |
| **Sex** |  |  | |  |  | |  |
| Male | _ | _ | |  | _ | | _ |
| Female | 1.18 (1.11-1.25) | <0.001*** | |  | 0.96 (0.80-1.14) | | 0.63 |
| **Ethnicity** |  |  | |  |  | |  |
| White | _ | _ | |  | _ | | _ |
| South Asian | 0.80 (0.63-1.02) | 0.07 | |  | 1.27 (0.82-1.97) | | 0.28 |
| **Smoking status** |  |  | |  |  | |  |
| Never smoker | _ | _ | |  | _ | | _ |
| Ex-smoker | 1.11 (1.03-1.18) | 0.004** | |  | 1.16 (0.82-1.63) | | 0.41 |
| Current smoker | 1.13 (1.04-1.23) | 0.005** | |  | 1.11 (0.91-1.35) | | 0.30 |
| **Disease duration (years)** |  |  | |  |  | |  |
| <5 | _ | _ | |  | _ | | _ |
| 5-9 | 1.73 (1.24-2.41) | 0.001** | |  | 0.52 (0.27-0.99) | | 0.047* |
| 10-14 | 2.56 (1.84-3.55) | <0.001*** | |  | 0.23 (0.12-0.44) | | <0.001*** |
| 15-19 | 3.16 (2.27-4.39) | <0.001*** | |  | 0.13 (0.07-0.25) | | <0.001*** |
| ≥20 | 3.43 (2.47-4.74) | <0.001*** | |  | 0.04 (0.02-0.09) | | <0.001*** |
| **Disease location** |  |  | | **Disease extent** |  | |  |
| Ileal | _ | _ | | *Proctitis* | _ | | _ |
| Colonic | 0.41 (0.37-0.45) | <0.001*** | | *Left-sided* | 0.82 (0.52-1.30) | | 0.41 |
| Ileocolonic | 0.85 (0.80-0.91) | <0.001*** | | *Extensive* | 1.09 (0.70-1.70) | | 0.70 |
| Exclusive upper GI | 0.71 (0.53-0.96) | 0.03* | |  |  | |  |
| **Disease behaviour** |  |  | |  |  | |  |
| Inflammatory | _ | _ | |  | _ | | _ |
| Penetrating | 3.06 (2.81-3.33) | <0.001*** | |  | _ | | _ |
| Stricturing | 2.70 (2.51-2.90) | <0.001*** | |  | _ | | _ |
| **Perianal involvement** | 0.99 (0.93-1.06) | 0.88 | |  | _ | | _ |
| **Medical therapy** |  |  | |  |  | |  |
| Corticosteroids | 0.96 (0.90-1.02) | 0.21 | |  | 0.79 (0.65-0.97) | | 0.02* |
| Thiopurines | 0.92 (0.85-0.99) | 0.04* | |  | 0.78 (0.63-0.96) | | 0.02* |
| Biologics | 1.02 (0.96-1.09) | 0.56 | |  | 0.58 (0.44-0.75) | | <0.001*** |
| * p <0.05, ** p <0.001, *** p <0.001  Adjusted for age at diagnosis, sex, ethnicity, smoking status, disease duration and disease extent | | | | | | | |
